# Neuroproteomics and Systems Biology Approach to Identify Temporal Biomarker Changes Post Experimental Traumatic Brain Injury in Rats

**DOI:** 10.3389/fneur.2016.00198

**Published:** 2016-11-22

**Authors:** Firas H. Kobeissy, Joy D. Guingab-Cagmat, Zhiqun Zhang, Ahmed Moghieb, Olena Y. Glushakova, Stefania Mondello, Angela M. Boutté, John Anagli, Richard Rubenstein, Hisham Bahmad, Amy K. Wagner, Ronald L. Hayes, Kevin K. W. Wang

**Affiliations:** ^1^Program for Neurotrauma, Neuroproteomics and Biomarkers Research, Department of Psychiatry, McKnight Brain Institute, University of Florida, Gainesville, FL, USA; ^2^Program for Neurotrauma, Neuroproteomics and Biomarkers Research, Department of Neuroscience, McKnight Brain Institute, University of Florida, Gainesville, FL, USA; ^3^Center of Innovative Research, Banyan Biomarkers Inc., Alachua, FL, USA; ^4^Department of Neurosurgery, Virginia Commonwealth University School of Medicine, Richmond, VA, USA; ^5^Department of Neurosciences, University of Messina, Messina, Italy; ^6^Brain Trauma Neuroprotection and Neurorestoration Branch, Center for Military Psychiatry and Neuroscience, Walter Reed Army Institute of Research, Silver Spring, MD, USA; ^7^NeuroTheranostics Inc., Detroit, MI, USA; ^8^Henry Ford Health System, Detroit, MI, USA; ^9^Department of Neurology, SUNY Downstate Medical Center, Brooklyn, NY, USA; ^10^Department of Physiology and Pharmacology, SUNY Downstate Medical Center, Brooklyn, NY, USA; ^11^Faculty of Medicine, Beirut Arab University, Beirut, Lebanon; ^12^Department of Anatomy, Cell Biology and Physiological Sciences, Faculty of Medicine, American University of Beirut, Beirut, Lebanon; ^13^Department of Physical Medicine and Rehabilitation, University of Pittsburgh, Pittsburgh, PA, USA; ^14^Safar Center for Resuscitation Research, University of Pittsburgh, Pittsburgh, PA, USA

**Keywords:** proteomics, traumatic brain injury, controlled cortical impact, biomarker, prognosis and therapeutics, inflammation, oxidative stress

## Abstract

Traumatic brain injury (TBI) represents a critical health problem of which diagnosis, management, and treatment remain challenging. TBI is a contributing factor in approximately one-third of all injury-related deaths in the United States. The Centers for Disease Control and Prevention estimate that 1.7 million people suffer a TBI in the United States annually. Efforts continue to focus on elucidating the complex molecular mechanisms underlying TBI pathophysiology and defining sensitive and specific biomarkers that can aid in improving patient management and care. Recently, the area of neuroproteomics–systems biology is proving to be a prominent tool in biomarker discovery for central nervous system injury and other neurological diseases. In this work, we employed the controlled cortical impact (CCI) model of experimental TBI in rat model to assess the temporal–global proteome changes after acute (1 day) and for the first time, subacute (7 days), post-injury time frame using the established cation–anion exchange chromatography-1D SDS gel electrophoresis LC–MS/MS platform for protein separation combined with discrete systems biology analyses to identify temporal biomarker changes related to this rat TBI model. Rather than focusing on any one individual molecular entity, we used *in silico* systems biology approach to understand the global dynamics that govern proteins that are differentially altered post-injury. In addition, gene ontology analysis of the proteomic data was conducted in order to categorize the proteins by molecular function, biological process, and cellular localization. Results show alterations in several proteins related to inflammatory responses and oxidative stress in both acute (1 day) and subacute (7 days) periods post-TBI. Moreover, results suggest a differential upregulation of neuroprotective proteins at 7 days post-CCI involved in cellular functions such as neurite growth, regeneration, and axonal guidance. Our study is among the first to assess temporal neuroproteome changes in the CCI model. Data presented here unveil potential neural biomarkers and therapeutic targets that could be used for diagnosis, for treatment and, most importantly, for temporal prognostic assessment following brain injury. Of interest, this work relies on *in silico* bioinformatics approach to draw its conclusion; further work is conducted for functional studies to validate and confirm the omics data obtained.

## Introduction

Traumatic brain injury (TBI) is a major health concern for which diagnosis, care management, and treatment remain challenging. Each year, approximately 1.7 million people sustain a TBI in the United States, out of which 52,000 die, 275,000 are hospitalized, and 1.365 million are treated and released from emergency care. Importantly, TBI is a contributing factor to a third (30.5%) of all injury-related deaths in the United States (1–3). TBI also accounts for a larger percentage of injuries and casualties among United States military personnel serving in Iraq and Afghanistan compared to previous conflicts, mainly due to the prevalent use of improvised explosive device and the high incidence of mild blast-overpressure exposure resulting in TBIs of various severities (4). The growing number of TBI cases among military personnel and civilians has brought new urgency to research efforts aimed at developing improved TBI diagnostic, prognostic, and treatment assays.

Neurological examination by the Glasgow Coma Scale (GCS) has been traditionally employed to assess acute TBI. However, this clinical tool has a number of limitations making it often inaccurate to categorizing TBI severity in patients (5). In most cases, clinical TBI status may be difficult to determine due to confounding medical treatments such as sedatives, opioids, or neuromuscular blocking agents (6, 7) and to illicit drug use and/or alcohol intake (8–10). The evolving nature of brain injury progression can further complicate GCS assessment. Current imaging methods such magnetic resonance imaging (MRI) and computer tomography (CT) provide more objective information on the magnitude and localization of the injury. However, CT scans lack the sensitivity to detect mild to moderate diffuse brain injury, and the availability and feasibility of MRI acutely limit their broad clinical application (11, 12). Taken together, these limitations and challenges illustrate the need for facile, rapid, and reliable alternate methods to assess brain injury.

Efforts to elucidate the complex mechanisms of TBI progression and seek sensitive and specific biomarkers that can aid in optimizing TBI prognosis at acute and chronic long-term time points have been the main focus in the field of brain neurotrauma. Biomarker research has produced several putative, candidate TBI markers that can be derived from brain tissue, blood, and cerebrospinal fluid (CSF). These biomarkers are often assessed *via* various immunoassays, such as Western blotting or enzyme-linked immunosorbent assay (ELISA). For instance, our group has examined the accumulation of spectrin and its calpain-cleaved breakdown products in the CSF and brain tissue following TBI (13–15). In addition, our recent studies using controlled cortical impact (CCI) model have demonstrated progressive brain pathologies in white matter involving myelin loss, delayed microvascular damage, and appearance of focal microbleeds that are temporally and regionally associated with punctate blood–brain barrier breakdown and upregulation of the glial and inflammatory biomarkers in the brain tissue starting from 24 h and progressing over 3 months following experimental TBI (16). S-100β, a calcium binding protein, has been used as well as severe TBI marker in serum (17–19) and CSF (20–22). Additionally, the concentration of myelin basic protein is elevated in human serum collected from pediatric TBI patients (18). Further, Tau proteins have been associated with elevated intracranial pressure, a symptom or component of TBI (23), and phosphorylated tau has been identified in serum up to several months after severe TBI (24). Nevertheless, despite the identification of these biomarkers *via* targeted approaches, many of them suffer from lack of TBI specificity and may not indicate TBI chronic temporal changes.

Recently, bioinformatics and in particular the application of neuroproteomic strategies to central nervous system (CNS) injuries has emerged as a promising biotechnology for identifying novel pathways and biological processes relevant to TBI pathophysiology, as well as pointing out which key genes/proteins may serve as potential biomarkers and therapeutic drug targets (25–29). The potential of neuroproteomics platforms have been explored using acute paradigms of TBI (26, 27, 29–35), spinal cord injury (36–44), and cerebral ischemia or stroke (34, 45–50). Our group has previously reported TBI effects upon the global proteome where we combined cyanine labeling with SDS PAGE–capillary LC–MS/MS to study hippocampal tissue (30). Results from this work provided a framework for subsequent rapid and comprehensive sequence-specific biomarker discovery strategies that are currently used in our laboratory. This strategy employs tandem strong cation–anion exchange chromatography (first dimension) followed by 1D gel electrophoresis (second dimension) prior to LC–MS/MS of tryptic peptides extracted from the gel. This “bottom up” protein identification revealed 59 differentially expressed proteins (of which 21 were decreased and 38 were increased) in cortical tissue collected 48 h after CCI in rats (26). Nonetheless, studying TBI at multiple time points by MS/MS-based proteomics remains crucial, as it enables the correlation of relative protein expression to disease progression in experimental TBI animal models, which can be translated to clinical outcome metrics.

In this present work, we employed the well-established rat CCI model to examine the global proteomic differences between acute (1 day post-CCI) and subacute (7 days post-CCI) TBI using the cation–anion exchange chromatography-1D SDS gel electrophoresis (CAX-PAGE) LC–MS/MS platform (26, 45). We then combined the temporal neuroproteomic response with systems biology strategies to infer time-dependent changes in cellular pathways caused by CCI. We also applied systems biology analyses to identify non-redundant pathways relevant to TBI at these different time points and conducted gene ontology analyses of the proteomics data to categorize the differentially expressed proteins by molecular function, biological process, and cellular localization. To the best of our knowledge, this study is among the first to assess the TBI protein dynamic changes associated with temporal resolution. Data from this work can be translated clinically to discover new potential biomarkers and therapeutic targets for TBI that could be used to better treat or prevent sequelae associated with this disease.

## Materials and Methods

### Animal Housing Conditions

Animal handling and processing were conducted in compliance with the National Institutes of Health Guidelines for Animal Research (Guide for the Care and Use of Laboratory Animals) and according to the laws of the USA and regulations of the Department of Agriculture. They were also approved by Institutional Animal Care and Use Committee (IUCAC) at the University of Florida. Rats were maintained and housed under pathogen-free conditions with constant temperature and humidity control.

### Implementation of *In Vivo* Controlled Cortical Impact in Rats

A digital electromagnetic CCI device (Benchmark™ Stereotaxic Impactor, MyNeurolab, USA) was used to model TBI in male Sprague-Dawley rats as described previously (16). A total of three groups were prepared with each group having seven rats. A total of 21 rats were divided into 3 groups, a craniotomy group, 1 day TBI, and 7 days TBI. For the TBI procedure, each rat was mounted in a stereotactic frame, and the right cortex (ipsilateral) was impacted with a 4-mm diameter impactor tip at a velocity of 3.5 m/s to a depth of 2.5 mm, at 1 day (*n* = 5) and 7 days (*n* = 5) post-CCI. Prior to surgery, rats were anesthetized (45 mg ketamine/kg, 5 mg xylazine/kg) by intraperitoneal injection. An adequate level of anesthesia was determined by monitoring the corneal reflex and withdrawal to painful stimuli for hind limbs. The injured rats were sacrificed by decapitation. From each group, we used five rats for our proteomics analysis, which has been validated to be statistically significant (26, 35, 51–53). For our control animals, sham-injured rats were subjected to identical surgical procedures but did not receive an impact injury.

### Brain Tissue Collection and Protein Extraction

Ipsilateral cortical brain tissue samples were rapidly dissected, washed with cold saline, sip frozen in liquid nitrogen then stored at −80°C until use. Brain tissues were pulverized into a powder over dry ice then solubilized in lysis buffer containing 1% (v/v) Triton X-100, 1 mM dithiothreitol (DTT), and one tablet protease inhibitor cocktail per 10 mL of total solution (Roche Diagnostics, IN, USA). The brain lysates were then centrifuged at 15,000 × *g* for 5 min at 4°C to remove insoluble debris. Protein concentrations were determined by detergent compatible (DC) protein assay (Bio-Rad, CA, USA).

### Cation–Anion Exchange Chromatography-1D SDS Gel Electrophoresis

A well-characterized *offline* multidimensional protein separation platform compatible with applying high throughput proteomic technology for biomarker identification was applied to this study according to the already published protocol (26, 27). In brief, this platform consisted of ion chromatography, which was composed of a sulfopropyl-strong cation exchange column (SCX-S1) and a quaternary ammonium-strong anion (SAX-Q1) modified sepharose prepacked ion-exchange columns (Bio-Rad). Purified cortical tissue protein lysates from each of the three groups (the craniotomy group, 1 day post-CCI, 7 days post-CCI) were pooled to achieve a requisite protein quantity of 1 mg per single CAX injection and to average inconsistent protein levels due to biological variability. Similar buffers and separation protocols were used as published (52, 53). UV chromatograms were collected at a wavelength of 280 nm for each run. Each 1-mL fractions was concentrated using Millipore YM-10 centrifugal filters (Millipore, MA, USA). Also, 20 μL of 2× Laemmli sample buffer (Invitrogen, NY, USA) was added to the YM-10 collection filters prior to collection by centrifugation at 1,000 × *g* for 3 min. For technical reproducibility, this experiment was repeated three times to compare each of the five control samples to their corresponding CCI samples (five samples for 1 day post-CCI and five samples for 7 days post-CCI). Protein concentration was measured afterward as a conformational step. Protein fractions were then run side-by-side (i.e., craniotomy fraction 1, CCI day 1 fraction 1, CCI day 7 fraction 1) on an 18-well, 10–20% gradient Tris–HCl Bio-Rad Criterion (Bio-Rad, CA, USA) gels for differential comparison of sham controls and the five CCI samples at each 1 and 7 days post-CCI. Gel bands were visualized and assessed for their intensity as described below.

### Gel Band Visualization and Densitometric Analysis

Gel bands were visualized by Coomassie blue staining (Bio-Rad, Hercules CA, USA). Quantitative densitometric analysis of select gel band intensities was performed using Image J software (Version 1.6, National Institutes of Health. Bethesda, MD, USA). Differential bands were manually identified and selected as regions of interest based on molecular weight estimation. The relative intensity in protein band fold change was derived by comparing intensity ratios among the sham control group, CCI day 1, and CCI day 7 and was calculated by dividing the greater value by the lesser value and adding a negative sign to indicate decrease after CCI. Any bands with fold change greater than 1.5 were selected for subsequent differential protein identification.

### Reversed-Phase Liquid Chromatography Tandem Mass Spectrometry

Nano-reversed-phase liquid chromatography tandem mass spectrometry (RPLC–MS/MS) was employed for protein separation and identification using the same parameters as recently published by our group (53). Nanoflow was performed on a NanoAcquity UPLC (Waters, Milford, MA, USA); the autosampler was used to load 2 μL of each sample onto a 5-μm particle size Symmetry 180 μm × 20 mm C18 trapping column at 4 μL/min for 10 min. Then, the sample plug was loaded onto a 1.7 μM particle size BEH130 C18 100 μm × 100 mm analytical column at 300 nL/min. The mobile-phase consisted of solvent A (water with 0.1% formic acid) and solvent B (acetonitrile with 0.1% formic acid). Separation was achieved within a run time of 115 min at a flow rate of 300 nL/min. The first linear gradient was from 1 to 40% B over 90 min; the second linear gradient was from 40 to 100% B over 5 min and held for 5 min before returning to initial mobile-phase composition (1%B). Tandem mass spectra were collected on LTQ-XL (Thermo, San Jose, CA, USA) using a data dependent acquisition method in Xcalibur 2.0.7 (Thermo), in which data-dependent scanning was specified as a criterion to select the top 10 most abundant ions using 11 separate scan events at a given chromatographic time point (115 min) for subsequent analysis. The mass spectrometer was set to perform a full-scan and subsequently MS/MS scans on the 10 most intense ions in the full-scan spectrum MS (scan event 1) with dynamic exclusion were enabled. Dynamic exclusion temporarily puts a mass into an exclusion list after its MS/MS spectrum is acquired, providing the opportunity to collect MS/MS information on the second most intense ion from the full-scan spectrum MS (scan event 1). All MS/MS spectra were analyzed using Proteome Discoverer 1.3 (Thermo), SEQUEST (version: 1.3.0.339), and X! Tandem [version: CYCLONE (2010.12.01.1)]. Database search engines were set up to search a trypsin-indexed uniprot-Rattus + norvegicus.fasta (accessed on 2015, 35,126 entries). The search was achieved using the average mass for matching the precursor with a fragment ion mass tolerance of 0.8 Da and a parent ion tolerance of 2.00 Da and a maximum of two missed cleavage sites. Carbamidomethylation (+57 Da) of cysteine was selected as a static modification, while the oxidation of methionine was selected as a dynamic modification. Using the output from SEQUEST and X! Tandem, Scaffold (version: Scaffold_3.3.3, Proteome Software) was used to validate, organize, and interpret mass spectrometry data. Peptide identifications were accepted if they could be established at greater than 95.0% probability as specified by the Peptide Prophet algorithm. Protein identifications were accepted if they could be established at greater than 99.9% probability and contained at least two peptides.

### Neurosystems Biology and Gene Ontology Analysis and Statistical Testing

Neurosystems biology analyses were conducted using the Elsevier’s Pathway Studio v. 10.0 (Elsevier, MD, USA) to construct non-redundant pathways relevant to the two different injury time points. This software was used to interpret biological meaning from gene (protein) expression, build and analyze pathways, and find relationships among genes, proteins, cell processes, and diseases as indexed by the ResNet database (54, 55). “Subnetwork Enrichment Analysis” (SNEA) algorithm was selected to extract statistically significant altered biological and functional pathways pertaining to each identified set of protein hits in our study. The algorithm compares the subnetwork distribution to the background distribution using one-sided Mann–Whitney *U*-test and calculates a *p*-value indicating the statistical significance of difference between two distributions.

Separate lists of International Protein Index (IPI) accession numbers of the identified upregulated and downregulated proteins for 1 and 7 days post-CCI were imported into the Pathway Studio. In each group, the upregulated proteins were assigned a value of +3, and the downregulated proteins were assigned a value of −3. The network analysis included a search for direct interactions and shortest paths between the identified proteins to map biologically relevant networks and identify relevant cellular processes. For a more comprehensive understanding of the classes of proteins found in the CCI model, we used the *PANTHER* bioinformatics analysis (http://www.pantherdb.org/genes/batchIdSearch.jsp) utilizing rat protein ontology database to classify proteins into distinct categories of molecular function and biological process. Integrated Venn diagram analysis was performed using the “InteractiVenn”: a web-based tool for the analysis of complex data sets (56).

## Results

### Combined CAX-PAGE

**Figure 1:**
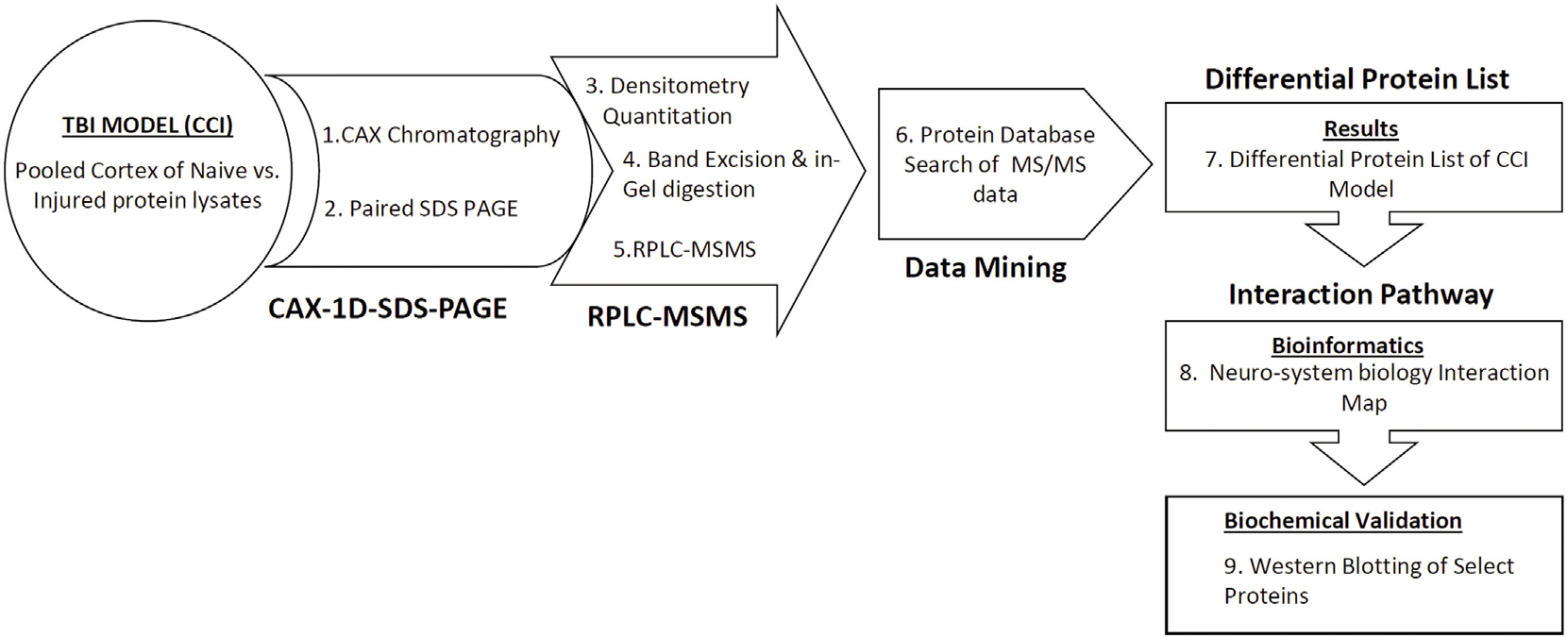
**Schematic diagram of the differential CAX-PAGE-LC–MS/MS proteomics platform used for the TBI study**. Proteins in the pooled extracts were resolved in two dimensions, first by cation–anion exchange chromatography followed by 1D-SDS-PAGE. Differential bands were selected, excised for in gel trypsin digestion, and analyzed by data dependent LC–MS/MS. Protein database search against a rat database generated a list of proteins with altered expression. The identified differential proteins were subjected to neurosystems biology pathway analysis.

**Figure 2:**
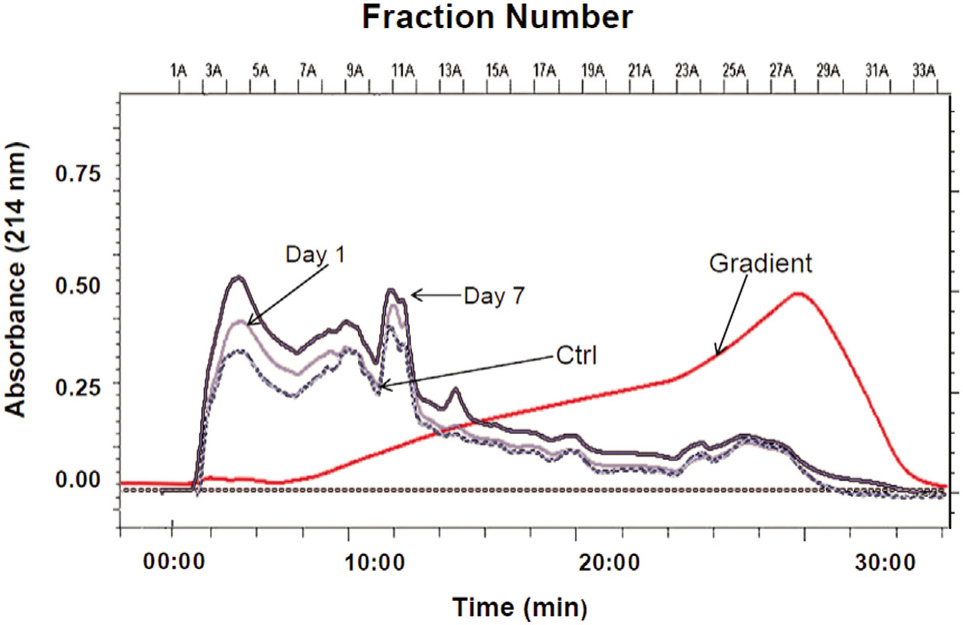
**CAX chromatographic separation of sham control and CCI (1 and 7 days)**. Overlaid CAX chromatograms of pooled rat cortical lysates (*n* = 5) are shown at 214 nm absorbance and the gradient profile. Thirty 1-mL fractions were collected from each pooled sample lysate for SDS-PAGE separation.

This study employed a previously established neuroproteomics approach applied to TBI studies by our group (13–15) (Figure 1). Our experimental design required pooled ipsilateral cortical lysates (*n* = 5) from control craniotomy and injured rats (1 and 7 days post-CCI). The proteins in each sample were resolved using two-dimensional CAX-PAGE. Samples from either (1) craniotomy control group, (2) 1 day post-CCI, or (3) 7 days post-CCI were sequentially separated by tandem strong cation and anion exchange chromatography according to protein charge. The overlaid CAX chromatograms of the sham control and the two injured groups illustrate differences among the three experimental groups particularly in intensity in some regions (Figure 2).

**Figure 3:**
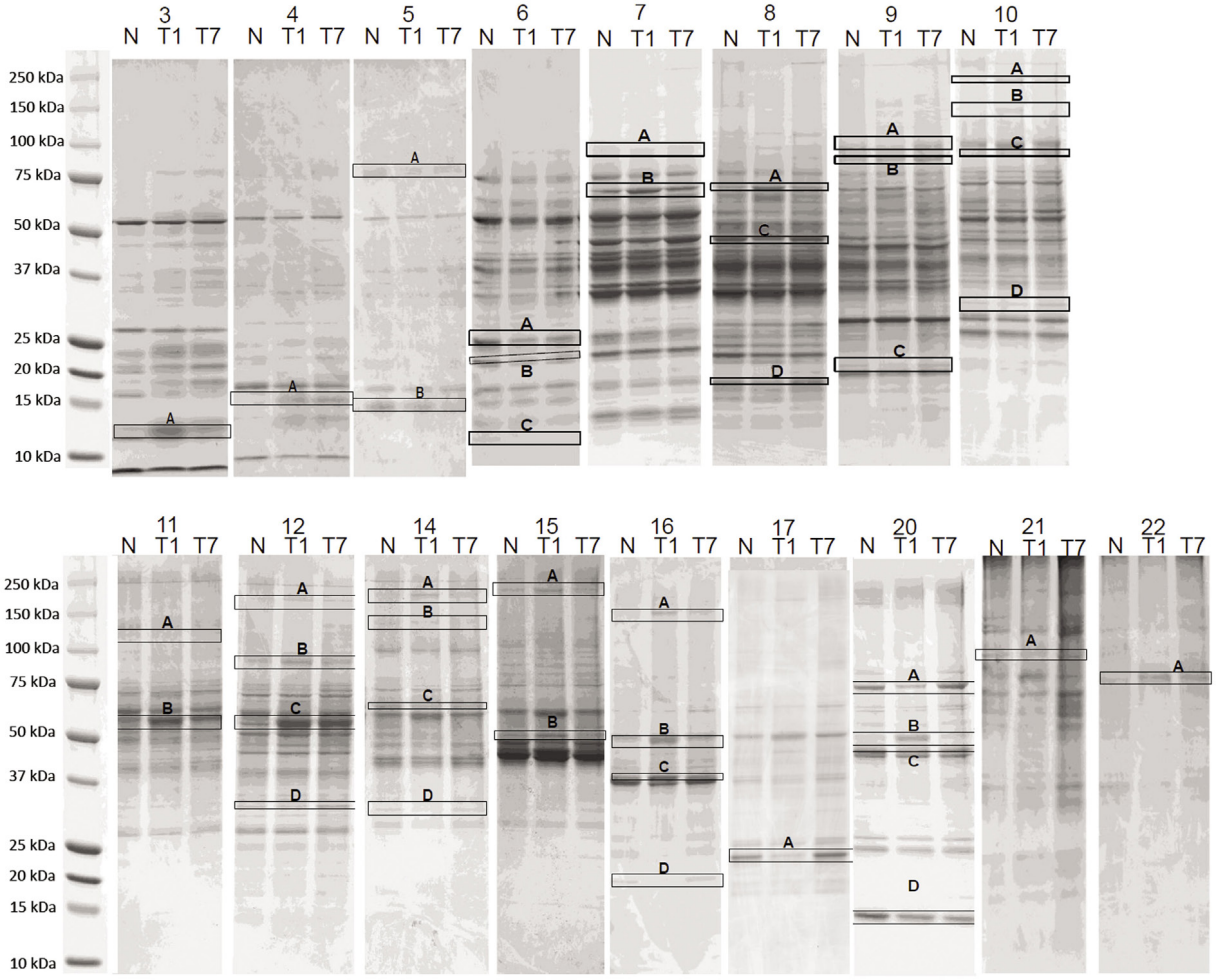
**Side-by-side SDS-PAGE separation of CAX fractions**. The 1-mL fractions collected from CAX separation were concentrated and loaded onto 1D-SDS-PAGE. Differential gel bands were selected (boxed and labeled) for trypsin digestion. In 1 day post-CCI, a total of 83 altered proteins were identified (47 upregulated vs. 36 downregulated) in the bands with fold change of 1.5 or more. In 7 days post-CCI, a total of 64 proteins were identified to be altered (47 upregulated and 17 downregulated) in the bands with fold change of 1.5 or more. *N* = Sham control, T1 = CCI day 1, T7 = CCI day 7. The numbers above each lane (topmost) are the CAX fraction number.

**Figure 4:**
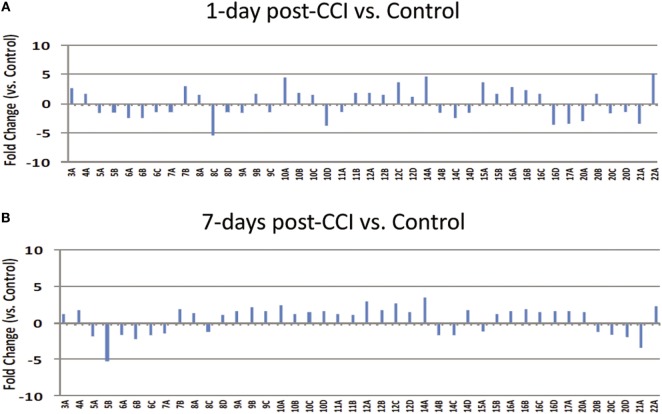
**Densitometric gel band analysis**. Differential band intensities of selected control and CCI bands were quantified using ImageJ densitometry software. **(A)** 1 day post-CCI and **(B)** 7 days post-CCI fold changes relative to the control. Forty gel bands were found to have more than 1.5-fold increase, and 30 gel bands were found to have more than 1.5-fold decrease in density.

Thirty, consecutive 1-mL fractions collected from each CAX separation were grouped (i.e., fraction 1 of sham control, fraction 1 of 1 day post-CCI, and fraction 1 of 7 days post-CCI) and loaded side-by-side onto a 1D gel to further resolve the proteins in the second dimension. A total of 42 differential bands were selected and excised from the gel for subsequent proteomic analysis as boxed and labeled in Figure 3. Relative fold changes were calculated between control and CCI (1 and 7 days) as shown in Figure 4, and bands with fold change greater than 1.5 were selected for subsequent differential protein identification. In the 1 day post-CCI group, half of the gel bands had decreased intensity and the other half of bands had increased intensity (Figure 4A). However, in 7 days post-CCI, 14 bands showed a decrease in intensity, and 28 gel bands showed an increase in intensity (Figure 4B).

### Identification of Differential Proteins by LC–MS/MS at 1 and 7 days Post-CCI

Following the gel densitometric analysis, 42 bands showing altered expression in either 1 or 7 days post-CCI were selected for LC–MS/MS analysis (Figure 3). Tables S1 and S2 in Supplementary Material summarize the identified proteins for each band with their corresponding peptide spectral data, sequence coverage and the directionality of the band intensities. In the 1 day post-CCI group, a total of 63 proteins were altered, of which 21 were only upregulated, 38 were only downregulated, and 4 were found to be identified in gel bands that expressed up/downregulation trends (Table S3 in Supplementary Material). While in 7 days post-CCI, a total of 56 proteins showed altered expression, with 41 proteins displaying increase in abundance alone, 13 proteins displaying decrease in abundance only, and 2 were found to demonstrate an increase and decrease in abundance at the same time (Table S4 in Supplementary Material).

### Unique and Common Upregulated and Downregulated Proteins at 1 and 7 days Post-CCI

In order to scrutinize the differences in protein expression at 1 and 7 days post-CCI, unique and common upregulated and/or downregulated proteins were identified between the two studied groups. There are 19 common proteins that showed an increase in abundance at both 1 and 7 days post-CCI (Table 1), including complement C3, peptidyl-prolyl cis-trans isomerase A (Pin1), elongation factor 2, protein kinase C and casein kinase substrate in neurons protein 1 (PACSIN), etc. Conversely, 15 common proteins were found to be downregulated at both time points 1 and 7 days post-CCI (Table 1), including superoxide dismutase (SOD), neurofascin (NF), calmodulin (CaM), etc.

**Table 1:** **Common upregulated and downregulated differentially altered proteins between the two different time points**.

Band	Identified proteins	Accession no.	Calc. M.W. (kDa)	Gel M.W. (kDa)	Gel band densitometry condirection	
					Day 1	Day 7
**(A) Upregulated proteins**						
4A	Peptidyl-prolyl cis-trans isomerase A (Pin1)*	IPI00387771	18	17	↑	↑
7B	Ba1-667	IPI00196656	107	75	↑	↑
9B	Elongation factor 2*	IPI00203214	95	87	↑	↑
10C	Elongation factor 2*	IPI00203214	95	85	↑	↑
9B	Cytoplasmic aconitate hydratase	IPI00207003	98	87	↑	↑
10A	Complement C3*	IPI00480639	186	200	↑	↑
12A	Complement C3 (fragment)	IPI00480639	186	200	↑	↑
10C	GMP synthase [glutamine-hydrolyzing]	IPI00372214	77	85	↑	↑
12B	Heat shock cognate 71 kDa protein	IPI00208205	71	80	↑	↑
12C	Dihydropyrimidinase-related protein 2	IPI00870112	62	55	↑	↑
12C	Isoform 2 of dihydropyrimidinase-related protein 3	IPI00203250	74	55	↑	↑
12C	Dihydropyrimidinase-related protein 5	IPI00331981	62	55	↑	↑
12C	Isoform M1 of pyruvate kinase isozymes M1/M2	IPI00231929	58	55	↑	↑
12C	WD repeat-containing protein 1	IPI00215349	66	55	↑	↑
14A	Alpha-1-inhibitor 3*	IPI00201262	164	220	↑	↑
16A	Alpha-1-inhibitor 3*	IPI00201262	164	145	↑	↑
16A	Isoform 1 of murinoglobulin-1	IPI00212666	165	145	↑	↑
16B	Serum albumin	IPI00191737	69	55	↑	↑
16C	Creatine kinase B-type	IPI00470288	43	40	↑	↑
16C	Protein kinase C and casein kinase substrate in neurons protein 1 (PACSIN)*	IPI00208245	50	40	↑	↑
16C	Gamma-enolase*	IPI00326412	47	40	↑	↑
22A	Brain acid soluble protein 1	IPI00231651	22	80	↑	↑
**(B) Downregulated proteins**						
5A	Aconitate hydratase, mitochondrial*	IPI00421539	85	85	↓	↓
7A	Aconitate hydratase, mitochondrial*	IPI00421539	85	85	↓	↓
5B	Superoxide dismutase [Mn], mitochondrial (MnSOD)*	IPI00211593	25	16	↓	↓
6B	Superoxide dismutase [Mn], mitochondrial (MnSOD)*	IPI00211593	25	23	↓	↓
6A	Triosephosphate isomerase*	IPI00231767	27	27	↓	↓
6B	Triosephosphate isomerase*	IPI00231767	27	23	↓	↓
6A	Gluthathione S-transferase (fragment)	IPI00231150	26	27	↓	↓
6A	Gluthathione S-transferase Mu1	IPI00231639	26	27	↓	↓
6B	Protein DJ-1*	IPI00212523	20	23	↓	↓
6C	Macrophage migration inhibitory factor (MMIF)*	IPI00230907	12	12	↓	↓
14B	1-phosphatidylinositol-4,5-bisphosphate phosphodiesterase beta-1	IPI00192534	138	140	↓	↓
14B	Ubiquitin-like modifier-activating enzyme 1	IPI00368347	118	140	↓	↓
14B	Isoform 1 of neurofascin (NF)*	IPI00206666	138	140	↓	↓
14C	Dihydropyrimidinase-related protein 2	IPI00870112	62	60	↓	↓
20C	Calreticulin	IPI00191728	48	52	↓	↓
20D	Calmodulin (CaM)*	IPI00231955	17	16	↓	↓
21A	Heat shock cognate 71 kDa protein	IPI00208205	71	70	↓	↓
21A	Isoform 1 of syntaxin-binding protein 1	IPI00205372	68	70	↓	↓

*(A) Nineteen proteins were identified to be commonly upregulated at 1 and 7 days post-CCI*.

*(B) Fifteen proteins were identified to be commonly downregulated at 1 and 7 days post-CCI*.

*Proteins of high interest relevant to our study.

Interestingly, some proteins were identified to be uniquely upregulated or downregulated at either 1 or 7 days post-CCI (Table 2). Indeed, 10 proteins were identified to be uniquely upregulated or downregulated at 1 day post-CCI (Table 2), while only 3 proteins were found to be exclusively upregulated at 7 days post-CCI (Table 2). Of those proteins showing an altered expression unique to day 1 or 7 are vinculin and protein-disulfide isomerase, which are differentially expressed at day 1, and annexin A5 (AnxA5), l-lactate dehydrogenase B chain (LDH-B), and thyroid hormone-binding protein μ-crystallin (CRYM) homolog, which are upregulated at day 7 only.

**Table 2:** **Unique upregulated and downregulated differentially altered proteins at each time point**.

Band	Identified proteins	Accession no.	Calc. M.W. (kDa)	Gel M.W. (kDa)	Gel band densitometry condirection	
					Day 1	Day 7
**(A) Unique altered proteins at 1-day post-CCI**						
3A	Peptidyl-prolyl cis-trans isomerase FKBP1A	IPI00231434	12	13	↑	−
3A	Zero beta-1 globin*	IPI00207146	16	13	↑	−
8A	Vinculin*	IPI00365286	117	75	↑	−
10B	Complement inhibitory factor H*	IPI00208659	140	150	↑	−
15B	Alpha-enolase*	IPI00464815	47	45	↑	−
8C	Alpha-enolase*	IPI00464815	47	50	↓	-
20B	Protein-disulfide isomerase (PDI)*	IPI00198887	57	55	↑	−
8C	Isoform mitochondrial of fumarate hydratase, mitochondrial	IPI00231611	54	50	↓	-
8D	Stathmin	IPI00231697	17	18	↓	-
8D	Cofilin-1	IPI00327144	19	18	↓	-
8D	Protein (peptidyl-prolyl cis/trans isomerase) NIMA-interacting 1 (predicted), isoform CRA_a	IPI00870306	18	18	↓	-
**(B) Unique altered proteins at 7 days post-CCI**						
12D	Annexin A5 (AnxA5)*	IPI00471889	36	29	−	↑
12D	l-lactate dehydrogenase B chain (LDH-B)*	IPI00231783	37	29	−	↑
12D	μ-crystallin homolog (CRYM)*	IPI00214448	34	29	−	↑

*(A) Ten proteins were identified to be uniquely upregulated or downregulated at 1 day post-CCI*.

*(B) Three proteins were identified to be uniquely upregulated at 7 days post-CCI*.

*Proteins of high interest relevant to our study.

### Temporal Network Analysis of Altered Proteins at 1 and 7 days Post-CCI

**Figure 5:**
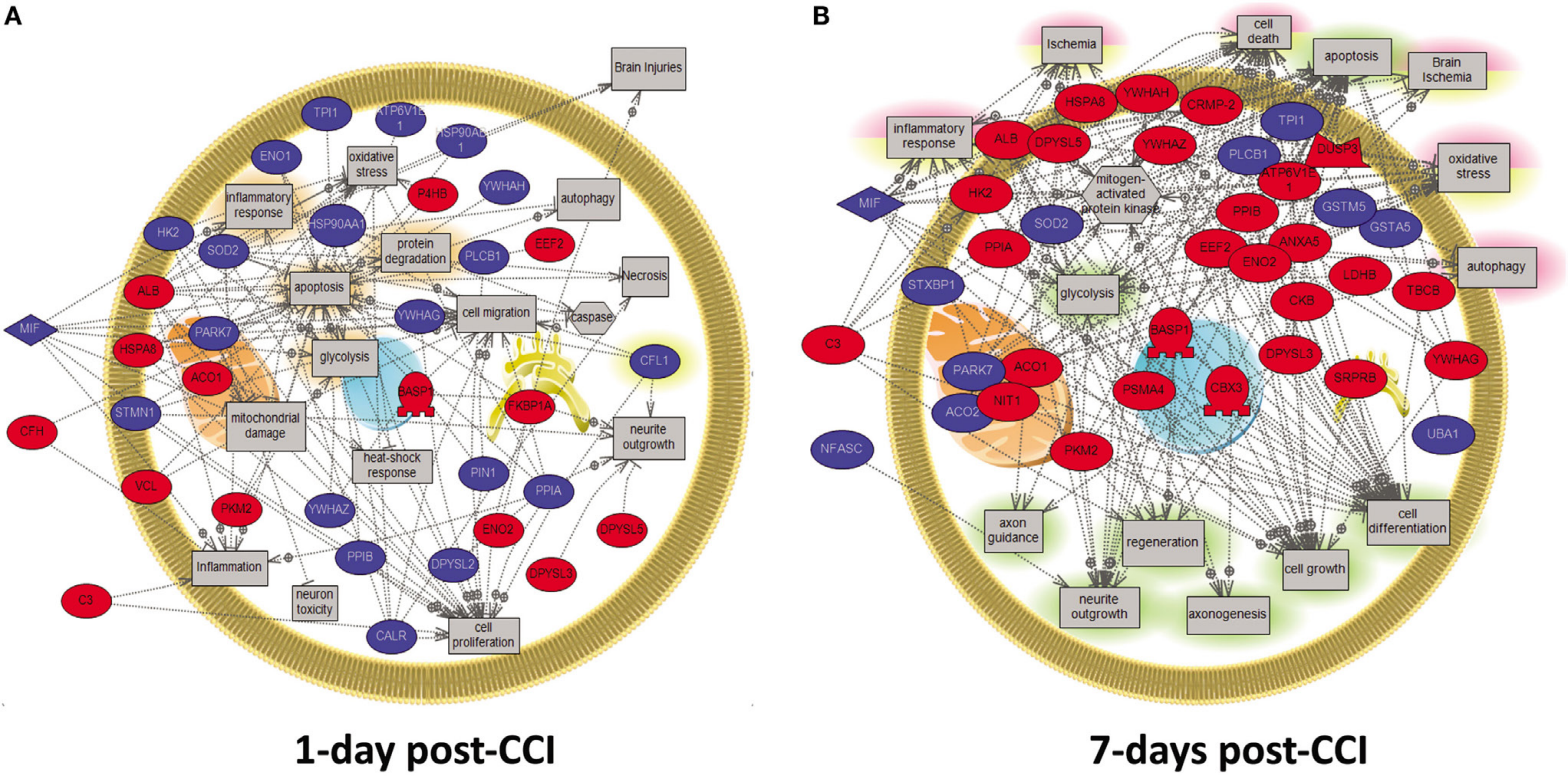
**Molecular and biological pathway interaction map analysis of altered proteins from the CCI samples (1 vs. 7 days post-CCI)**. Using Pathway Studio 9.0, altered proteins relevant to CCI at different time points were analyzed. The network was generated using “direct interaction” algorithm to map biological processes and interactions among altered proteins. Several processes believed to be central to the pathogenesis of CCI were identified using this search. **(A)** Examples of those processes including apoptosis, inflammatory response, oxidative stress, and autophagy were shown to be altered at day 1. **(B)** Similarly, at day 7, processes including apoptosis, inflammatory response, oxidative stress, ischemia as well as cell regeneration and cell growth were shown to be altered. The red color represents upregulated proteins, while the blue color signifies proteins that are downregulated.

To further understand the difference in protein expression at the cellular level post-CCI between 1 and 7 days, analysis of the specific enriched pathways was performed. The analysis of neuroproteomics data generated interaction maps as presented in Figure 5. The network was generated using the “direct interaction” algorithm to map cellular process and interactions between altered proteins. The red color represents upregulated proteins in day 1 injury onset, while the blue color shows proteins that are downregulated. In the 1 day post-CCI group, the majority of the proteins are associated with apoptosis, inflammatory response, oxidative stress, and autophagy (Figure 5A). The proteins identified in the 7-day post-CCI samples were also found to be involved not only in apoptosis, inflammatory response, and oxidative stress but also in ischemia as well as cell regeneration and cell growth (Figure 5B).

### Gene Ontology Analysis of the Differentially Expressed Proteins at 1 and 7 days Post-CCI

**Figure 6:**
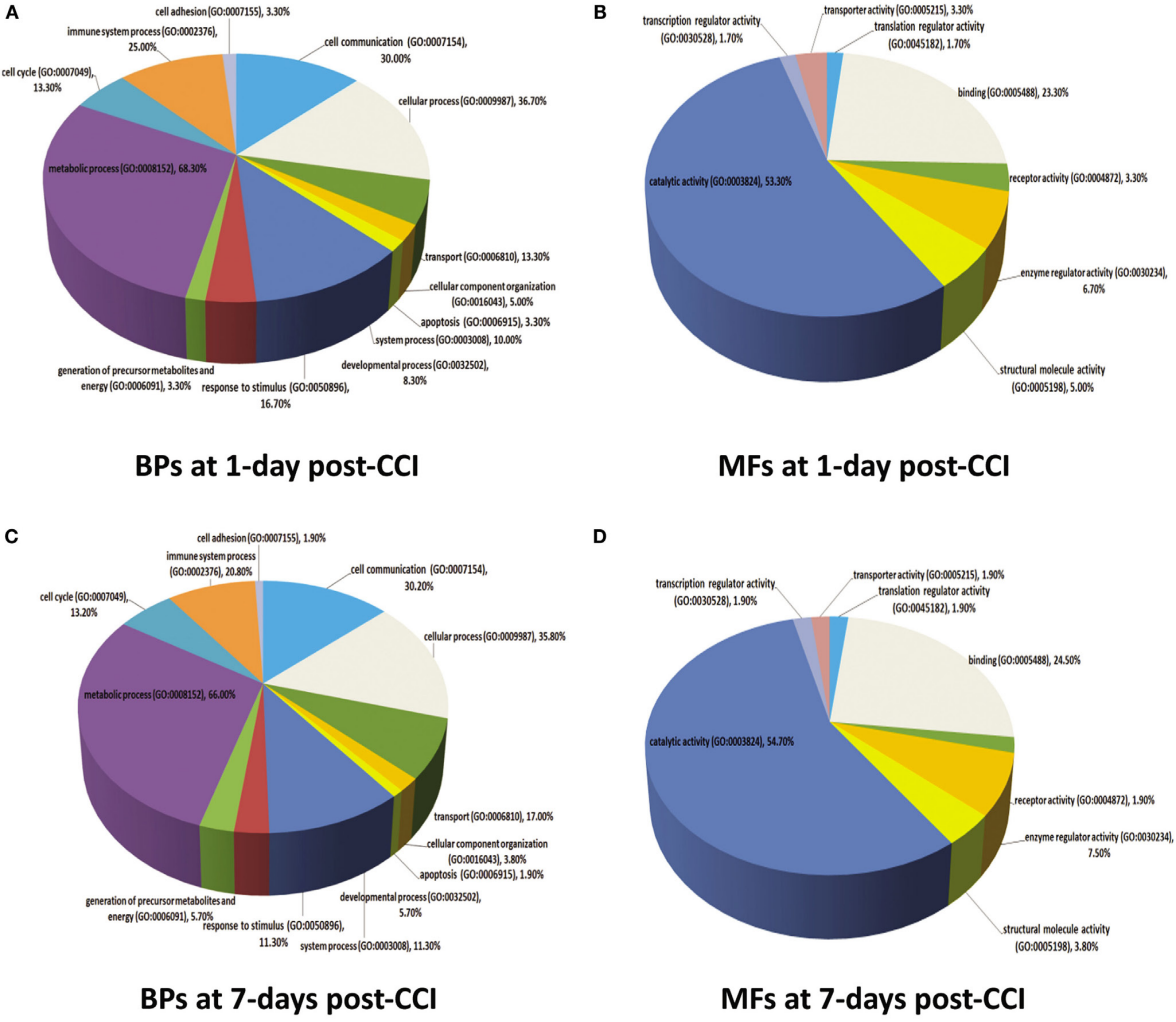
**Molecular functions (MFs) and biological processes (BPs) depicting proteins that are altered in response to CCI at 1 and 7 days are shown in pie charts**. Proteins are classified into families and subfamilies of shared function, which are then categorized using a highly controlled vocabulary (ontology terms) by biological process **(A,C)** and molecular function **(B,D)**. At 1 day post-CCI, 59 assignments were obtained for biological process and were sorted into 13 categories **(A)**, while 142 molecular functions obtained were arranged into 8 classifications **(B)**. At 7 days post-CCI, 52 assignments were obtained for biological process and sorted into 13 classifications **(C)**, whereas 119 molecular functions obtained were sorted into 8 categories **(D)**. Some proteins may be assigned for more than one molecular function and biological process. The percentages listed are calculated as the number of proteins associated with a particular functional block normalized to the total number of proteins.

For a more comprehensive understanding of the classes of proteins found in the different brain injury samples (1 vs. 7 days), we used the PANTHER application for the rat protein ontology database to classify proteins into distinct categories. Data were defined by biological processes and molecular functions (Figure 6). For 1 day post-CCI, 59 assignments obtained for biological processes were sorted into 13 classifications (Figure 6A), while 142 molecular functions were sorted into 8 classifications (Figure 6B). Similarly, for 7 days post-CCI, 52 assignments obtained for biological processes were categorized into 13 classifications (Figure 6C), whereas 119 molecular function were sorted into 8 classifications (Figure 6D). The classification categories exceeded the number of proteins, and this is due to the fact that some proteins may be assigned more than one molecular function and biological process. Percentages listed represent the number of proteins linked with a particular functional block normalized to the total number of proteins at each time point. Interestingly, the same biological processes and molecular functions were enriched at 1 and 7 days post-CCI but with differences noted in percentages of proteins corresponding to each functional block.

## Discussion

Traumatic brain injury imposes a substantial public burden where diagnosis, management, and treatment remain challenging. With an estimated 10 million people affected annually by TBI worldwide, it is predicted by the World Health Organization (WHO) that by the year 2020, TBI will surpass many diseases to become the third leading cause of global mortality and disability (57). Efforts have been made and continue to focus on elucidating the complex molecular mechanisms underlying TBI pathophysiology and defining specific biomarkers for this disease. Recently, bioinformatics and in particular neuroproteomic studies have proven to be a contemporary and convenient tool in biomarker discovery for many human diseases including CNS injury. In this study, we employed our rat CCI model to decipher mechanistic changes underlying TBI at the level of the proteome after 1 day (acute) and, for the first time, 7 days (subacute) following TBI injury.

The high throughput nature of proteomic studies generates tremendous amount of data. Systems biology has been considered latest contemporary domain in biological science that aims for a system-level elucidation of complex biological processes (58). Rather than focusing on the individual molecular entity, systems biology seeks to understand the system dynamics that govern protein networks and the functional set of proteins that regulate decisions related to the disease or injury.

In our study, the multidimensional CAX-PAGE RPLC–MS/MS proteomic platform was utilized to identify proteome changes in acute (1 day post-CCI) and subacute (7 days post-CCI) TBI. This study provides an assessment of the global temporal changes in protein expression in contrast to our earlier single time point TBI proteomic study at 48 h post-trauma (26). Similar to the latter study, the altered TBI proteome is contrasted against the sham cortical proteome. A total of 63 proteins were altered in acute TBI, where 21 were upregulated, 38 were downregulated, and 4 were upregulated and downregulated simultaneously. On the other hand, 56 proteins were identified to have changed in abundance in subacute TBI, with 41 proteins showing increase in expression, 13 proteins showing decrease in expression, and 2 proteins revealing increased and decreased expression together. The total number of altered proteins in this study is higher compared to our previous study, where a total of 66 proteins were identified at 1 and 7 days post-CCI, while 59 proteins were identified at 48 h post-TBI in the cortical tissue. Proteins with altered expression following TBI were either upregulated or downregulated in response to injury. Here, systems biology approach was utilized to identify altered cellular processes and pathways associated with the differential proteome of acute and subacute TBI.

There are 15 proteins showing decreased abundance in both acute and subacute TBI (Table 1B). Among these are proteins such as macrophage migration inhibitory factor (MMIF), aconitate hydratase (aconitase), SOD, NF, and CaM. MMIF is a brain inflammatory mediator that has been shown to be elevated 1 day post-TBI (59). Although it is also increased in Alzheimer’s disease (AD) and mild cognitive impairment (60), our results showed that MMIF expression is decreased at 1 and 7 days post-CCI. This may contribute to the presence of other downstream proteins regulating MMIF function in the brain. Aconitate hydratase (aconitase) was also shown to be downregulated at acute and subacute time points in this present work. Studies reveal that loss of cellular aconitase activity can been used as an index of oxidative stress damage (61, 62).

Superoxide dismutase is another oxidative stress-related protein. It is present in very small amounts in the extracellular matrix to remove excess superoxide anions that are released following TBI. Experiments performed on rats with cerebral contusion have shown that administration of exogenous lecithinized SOD reduces edema produced by superoxide ions post-TBI (63, 64). In our study, SOD was downregulated (nearly twofold change) at 1 day post-CCI, with a greater decrease in expression of around fivefold change at 7 days post-CCI. This is consistent with a previous study done on mice with brain injury, where SOD levels were significantly lower in the TBI group compared with the sham group (65). Interestingly, SOD may serve as a therapeutic target in TBI, where studies assessing the potential protective effect of SOD2 following brain injury have shown that ameliorating its expression in mice exhibited enhanced neuroprotection and decreased oxidative stress (65, 66). Also, the overexpression of manganese superoxide dismutase (MnSOD) protected tissues from radiation by reducing experimental inflammation (67), which can be further tried in TBI.

Additionally, CaM, a Ca^2+^ sensor, which showed decrease in expression in acute and subacute TBI, is known to mediate critical signaling functions through binding and regulating a diverse population of downstream targets referred to as calmodulin-binding proteins (CaMBPs). In TBI, our group developed a novel proteomic method that identified a total of 69 potential CaMBPs, of which 26 were known CaMBPs and 43 were putative novel CaMBPs (68). A study evaluating the biochemical mechanisms contributing to memory loss post-TBI have showed that unselective activation of calcium/calmodulin-dependent protein kinase II (CaMKII) signaling may disrupt the machinery for memory formation, resulting in this memory loss (69). Another study on calcineurin, a calcium/calmodulin-dependent phosphatase, has also found that its isoforms are differentially modulated by acute TBI in rats in a regionally specific manner (70). Future studies can be hence employed to further assess exactly the functions of CaM downstream targets in TBI, which may help improve current therapeutic interventions and/or develop new treatments.

On the other hand, a total of 19 proteins showed increase in abundance in both acute and subacute TBI samples (Table 1A). Those include complement C3, Pin1, elongation factor 2, and PACSIN. The complement component C3 was found to be upregulated at both 1 and 7 days post-CCI, with higher expression at 7 days. This protein is necessary for normal neutrophil extravasation comprising the inflammatory reaction in the brain following TBI (71). Also, it has been suggested that complement activation does not induce membrane-damaging effects in penetrating TBI (72) but rather C3-positive microglia and co-localization of both C3 and membrane-attack complex were found on cell bodies and axons of neurons at the TBI areas in rats (73). Thus, C3 may serve as a potential therapeutic target modulating the CNS inflammatory injury (71).

In addition, Pin1 enzyme was highly expressed at both time points in our study. This protein has a neuroprotective role in the CNS where its ablation leads to premature aging and neurodegeneration. Patients with AD were found to have inactivated Pin1 in their neurons (74–76). Besides, studies have revealed that polymorphisms of the *PIN-1* gene may affect neurodegeneration in the hippocampal area, which may lead to mild cognitive impairment and eventually AD (77, 78). Hence, relevance of Pin1 pathology to TBI may be taken into consideration in the future to assess its potential role in TBI-induced dementia and AD especially that many posttranslational modifications, which may follow protein synthesis, are similar between AD and TBI (79).

Another protein with increased abundance is PACSIN. Although its function in TBI is still unclear, a study has revealed the involvement of PACSIN in signal transduction to the cytoskeleton of neurons through phosphorylation by protein kinase C and casein kinase 2 (80–82). Importantly, this phosphorylation of casein kinase 2 has been proven to precipitate Rac1-dependent spine formation in dendrites of hippocampal neurons (83). Moreover, PACSINs play crucial roles in protein packing and tabulation activity through tip-to-tip and wedge loop-mediated lateral interactions on the surface of neuronal membranes (84), and they promote biogenesis of membrane-trafficking intermediates by engaging with inositol 5-phosphatase (OCRL1) (85). Besides, a proteomic analysis of the hippocampus in early-stage AD model mice has revealed significant changes in the levels of 14 proteins, one of which is PACSIN 1 that was significantly increased (86). This consistency in the levels of certain proteins, such as PACSIN, between TBI and AD, points to a common mechanistic ground linking the pathophysiology of both maladies (79). More than that, many of the neuroprotective proteins that are overexpressed in TBI, with higher expression in subacute TBI, can serve as novel candidate biomarkers and potential targets for TBI in the future.

Some of the altered proteins identified in our proteomic analysis were unique to each time point as well, where 10 proteins were recognized at 1 day post-CCI alone, while 3 were unique to 7 days post-CCI time point. Of the proteins that are altered at 1 day post-CCI only are vinculin and protein-disulfide isomerase (Table 2A). Vinculin, an integrin complexing protein known to be associated with synaptic destabilization and process retraction, has been shown to dissociate from cellular membranes following TBI (87). It usually participates in anchoring actin filaments to the cell membrane and plays a crucial role in cell motility. In a study done to investigate the levels of actin-anchoring proteins vinculin, talin, and paxillin in rat brains following stab wound injury, it has been shown that astrocytes in the white matter stained positive for these proteins, while none of them was found in the cortex (88). In our study, vinculin was upregulated at 1 day post-CCI but not expressed after 7 days. Moreover, protein-disulfide isomerase, a stress protein that is overexpressed in response to hypoxia in primary-cultured glial cells, can have beneficial effects against brain ischemia and plays a critical role in resistance to ischemic damage through its effect against apoptotic cell death (89). It is also upregulated in acute TBI alone in our study. Also, three proteins were uniquely upregulated at 7 days post-CCI including AnxA5, LDH-B, and CRYM (Table 2B), which may have potential roles following injury and can serve as novel biomarkers for subacute TBI. AnxA5 induces neuronal membrane repair *via* the formation of a protective 2D bandage at the membrane damaged site (90). On the other hand, the increase in the levels of l-lactate dehydrogenase A chain (LDH-A) and decrease in LDH-B have been shown to contribute to high brain lactate levels, which are predictive of aging phenotypes (91). Finally, CRYM and thyroid hormone levels may be key factors in the development of the pipecolate pathway in the brain (92), especially that neonatal decrease in these hormones is well-known to influence brain development and cause mental retardation and neurological damage (93, 94).

Interestingly, our proteomics data analysis also revealed alteration in a subset of proteins that have not been previously studied or tackled in TBI. For instance, at both 1 and 7 days post-CCI, a number of proteins were found to be upregulated including elongation factor 2, alpha-1-inhibitor 3, and gamma-enolase (Table 1A). Eukaryotic elongation factor 2 is known to play a key role in regulating the protein translational machinery and controlling ribosomal movements across the mRNA, thus affecting the survival of neurons in cases of elevated oxidative stress (95). Alpha-1-inhibitor 3, a protein of the alpha-macroglobulin family and an acute phase reactant protein, has been previously studied and shows a role in the early stages of the inflammatory response (96), yet it has never been studied in TBI. In addition, gamma-enolase, also known as neuron-specific enolase, possesses neuroprotective effects on cultured neurons from embryonic rat brain (97). On the other hand, a subset of proteins was found to be downregulated at both time points including triosephosphate isomerase, protein DJ-1, and isoform 1 of NF (Table 1B). Inhibition of triosephosphate isomerase was shown to induce neuronal death in cultured murine cortical cells, while protein DJ-1 possesses a neuroprotective role in Parkinson’s disease (98). The importance of those proteins is that they may serve as potential biomarkers and therapeutic targets for TBI, where future studies may be conducted to assess their exact function in the context of TBI. Moreover, other proteins that were found to be uniquely altered at each time point, such as alpha-enolase, complement inhibitory factor H, and zero beta-1 globin, and have never been studied in TBI, can also be assessed for their function in TBI (Table 2).

In order to scrutinize the differences in protein expression at a functional level, further analysis was carried forward using PANTHER software to identify enriched pathways and biological processes altered in TBI at different time points between acute and subacute states. This software is a unique resource that classifies genes and proteins by their functions using published scientific experimental evidence and evolutionary relationships abstracted by curators with the goal of predicting function even in the absence of direct experimental evidence. Proteins are classified into families and subfamilies of shared function, which are then categorized using a highly controlled vocabulary (ontology terms). In our study, common pathways were altered including apoptosis, inflammatory response, oxidative stress, and autophagy both at 1 and 7 days post-CCI. Yet, it is worthwhile to state that several cellular processes encompassing neuroprotective proteins were found to be enriched in subacute TBI, including cell regeneration, neurite outgrowth, axonogenesis, and cell growth. Interestingly, some pathways were found to be altered uniquely at 1 day post-CCI, such as cell migration, caspase, mitochondrial damage, neuron toxicity, and heat shock response, while others were found to be solely altered at 7 days post-CCI, including regeneration, axon guidance, axonogenesis, cell growth, and cell differentiation (Figure 5).

## Study Limitations

This work builds on previous and current recent studies from our and other labs that have showed the capabilities of neuroproteomics in identifying putative markers of TBI (UCH-L1, synaptotagmin, and spectrins) utilizing the same CAX separation platform as the one applied here (26, 27, 52). Several of these markers have been patented and been translated to clinical settings such as UCH-L1 and GFAP (19). However, this work has a number of limitations that relates to the study experimental design, methodology, and finally complying with the recent recommendation of the NIH common data elements (CDEs) for preclinical TBI (99).

First, our work was based on an *in silico* bioinformatics approach lacking validation or confirmation steps *via* wet lab techniques; these experiments were kept for the follow-up functional analysis study where we selected few of the identified proteins to study their dynamic alteration. Second, our work has considered only two time points (1 vs. 7 days), while in reality, longer time points post-injury should be assessed to give a comprehensive overview of protein changes. Third, this work assessed only cortical regions, while the emphasis now is to look at different brain regions that can depict the global injury profile rather than being biased to the cortical areas. Therefore, future studies including multiple regions such as the hippocampus and thalamus will provide a better insight of the injury profile. Besides, this work is using the CCI model, which may not be truly the ideal TBI model to recapitulate the different TBI pathologies. This will be gained in future studies through comparing our results to different models such as repetitive mild TBI, and consequently, analyzing clinical samples may be considered. It is likely that there will be common proteins/pathways retrieved; yet there also may be unique findings between focal, diffuse, or even axonal injury pathologies. As per the recommendation of the NIH CDEs for preclinical TBI, the use of female cohort should be included to highlight the diversity in injury profile and avoid any experimental bias; however, one should bear in mind that these neuroproteomics studies have their complexities and limitations in determining the number of samples to be used, the pooling consideration, and finally the time and cost they require. Finally, it is of interest to mention and highlight the shortcomings of this proteomics and other high throughput approaches (MRI, transcriptomics, gene arrays, etc.) generating “big data,” which may be biased toward the instrumentation in use or the method of selection. This will often lead to the missing of big chunk of extremely valuable data from any experimental study. To illustrate this point, it is estimated that the human genome contains around 20,000 protein-encoding genes, while the total number of proteins in human cells is estimated to range between 250,000 and one million. Nevertheless, any genomics study and/or complementary proteomics platform will identify on average 100–400 changed genes/proteins, which raises the question of the fate of other altered genes/proteins that have missed due to the methodology limitation. To conclude, this work utilizing systems biology concept has showed that it can highlight on several differential pathways and proteins, which can be of high prognostic and diagnostic value. Several of the identified protein hits can be translated clinically as putative markers in clinical TBI.

## Conclusion

As noted from the experimental model and results obtained, the aim of this work is to provide an overview of the altered protein dynamics that are commonly and/or uniquely altered in the acute and subacute phases of experimental model of TBI. Collectively, data obtained from this work suggest that many of the differentially identified proteins in the acute and subacute phases of TBI may serve as potential neural biomarkers and therapeutic targets for TBI. Of interest, the identification of the acute phase protein C3 seems to be a promising candidate marker that is easily measured in human TBI biofluids. Recently, there has been increased interest in the inflammatory markers in neurotrauma that can be coupled with neural specific protein to constitute a panel of TBI markers; several of these inflammatory markers have shown to be elevated clinically after severe TBI (100). Similarly, identified structural proteins constitute an interesting set of markers to be assessed post-injury time points, since these protein families have been among grass root of acute TBI biomarkers (βII–αII spectrins, ankyrins) (101–104).

## Author Contributions

KW, RH, and FK designed, developed, and tested the algorithm on real data (Mouse Genome). JG-C, ZZ, and AM prepared and performed the proteomics data, analyzed the omics, and interpreted the results. JA, RR, and AW helped in writing the manuscript. FK and HB assisted in the systems biology data analysis. OG has performed the TBI animal injury and sacrifice. All the authors assisted in the final assessment of data and reviewed the manuscript. AW, RH, and KW conceived the study design and obtained funding for the study. AB, SM, JG-C, AW, and ZZ revised and edited the manuscript. All the authors have read and approved the final manuscript.

## Conflict of Interest Statement

Dr. RH and KW own stock in Banyan Biomarkers Inc. Drs. FK, ZZ, JG-C, and SM were employees and received salaries from Banyan Biomarkers, Inc. The other authors declare no competing financial interests. The opinions stated are the private views of the authors and are not to be construed as official views of the Department of the Army or the Department of Defense.

## Abbreviations

AD, Alzheimer’s disease; AnxA5, annexin A5; CaM, calmodulin; CaMBP, calmodulin-binding proteins; CAX-PAGE, cation–anion exchange chromatography-1D SDS gel electrophoresis; CCI, controlled cortical impact; CNS, central nervous system; CRYM, thyroid hormone-binding protein μ-crystallin; LDH-B, l-lactate dehydrogenase B chain; MMIF, macrophage migration inhibitory factor; NF, neurofascin; PACSIN, protein kinase C and casein kinase substrate in neurons protein 1; Pin1, peptidyl-prolyl cis-trans isomerase A; RPLC–MS/MS, reversed-phase liquid chromatography tandem mass spectrometry; SOD, superoxide dismutase; TBI, traumatic brain injury.
